# Eastern king prawn *Penaeus plebejus* stock enhancement—Genetic evidence that hatchery bred prawns have survived in the wild after release

**DOI:** 10.3389/fgene.2022.975174

**Published:** 2022-11-08

**Authors:** H. K. A. Premachandra, Alistair Becker, Matthew D. Taylor, Wayne Knibb

**Affiliations:** ^1^ Centre for Bioinnovation, University of the Sunshine Coast, Sippy Downs, QLD, Australia; ^2^ New South Wales Department of Primary Industries, Port Stephens Fisheries Institute, Nelson Bay, NSW, Australia

**Keywords:** stock enhancement, genetic structure, *Penaeus plebejus*, eastern king prawn, mitochondrial control region

## Abstract

Eastern king prawn (*Penaeus plebejus*) is endemic to eastern Australia and is of high commercial and recreational value. As part of a recreational fisheries enhancement initiative, hatchery reared juveniles from Queensland were released into two, more Southern New South Wales (NSW) estuaries between 2014 and 2015. Responsible stock enhancement programs rely on knowledge of the population structure of the released species. Previously, in consideration of fisheries data, it was assumed the king prawn populations in Australia are one single breeding stock. In the present study, our first aim was to test this posit of no genetic differentiation using mtDNA control region (mtCR) sequences from the wild samples collected from four estuaries ranging from Queensland/NSW border (source of the stocked animals) to Southern NSW. The second objective was to test for signals of hatchery-released animals in the two stocked estuaries. All four surveyed populations had an extremely high level of haplotype diversity (average *h* = 99.8%) and low level of haplotype sharing between populations. Estimates of PhiPT values were <0.01 or close to zero and AMOVA test did not indicate any significant differences among populations. Further, phylogenetic analysis and principal coordinate analysis did not support division of samples by population. Collectively these results suggest that eastern king prawn populations along the NSW coast can be considered as a single stock and stocking from the Queensland samples will not necessarily impact the genetic composition of the overall stock. After stocking of two estuaries, sharing of haplotypes was moderate to very high in the stocked sites (>80% in some collections) but negligible in the two unstocked estuaries (≤2%, which is assumed to be background coancestry unrelated to the hatchery). Moreover, some haplotypes present in the hatchery broodstock were detected in stocked sites, but not in unstocked sites. The highest stocking signal was detected in the estuary which becomes isolated from the sea by sand barrier suggesting such “lakes” maybe more favourable for stocking than estuaries directly open to the sea. Findings in the current study should assist in designing and implementation of future prawn stocking programs.

## 1 Introduction

Eastern king prawn *Penaeus plebejus* is a high value species in commercial and recreational fisheries and an endemic species to the eastern waters of Australia, ranging from a northern limit at Swain Reefs in Queensland down to Northeast Tasmania in the South (Ruello, 1975; Montgomery et al., 2007). The species has a complex life history involving an adult Northerly migration to spawning grounds in Northern New South Wales (NSW) and Southern Queensland, and Southward dispersal of spawned larvae along a Western boundary current (East Australian Current), followed by recruitment into estuarine nursery habitats (Montgomery, 1990). Here they form aggregations which are targeted by recreational anglers, establishing a socially and economically valuable niche recreational fishery (Reid and Montgomery, 2005; Taylor, 2017). However, many NSW estuaries are closed to the ocean for periods sometimes extending to years due to the longshore movement of sand (Roy et al., 2001). As a result, many estuaries are recruitment limited and these estuaries have been identified as potential locations for the fisheries enhancement programs involving the release of hatchery-reared post-larvae.

Aquaculture-based enhancement is a management approach primarily involving the release of cultured/hatchery-bred individuals into nursery habitats to enhance the existing wild stocks, and has been identified as a useful fisheries management tool over the past decades (Blankenship and Leber, 1995; Molony et al., 2003; Taiarui et al., 2019). These enhancements are usually either stock enhancement (where releases are intended to both enhance catch and contribute to spawning stock), sea ranching (where the primary objective of release is improved catch) or restocking (where stock rebuilding is the principal goal). Stock enhancement programs internationally often try to avoid deleterious genetic effects, such as a reduction of the genetic diversity of the wild stocks (which may reduce future adaptability) and also possible inbreeding of released siblings (which could lead to inbreeding depression) (e.g., Blankenship and Leber, 1995; Taylor et al., 2005; Lorenzen et al., 2010). Assessment of the genetic structure of the existing wild populations and the hatchery stocks is typically integral to responsible enhancement programs.

Prawn releases have occurred for over 50 years in various countries, at large scales in China and Japan, but also in Kuwait, United States, Taiwan, Australia and Sri Lanka (Davenport et al., 1999; Loneragan et al., 2006; Setio, 2016) resulting in varying levels of success (Kitada, 2018; Serajuddin et al., 2018; Kitada, 2020). Highly variable and low recapture rates were reported from several prawn stock enhancement programs in Japan where in the majority of cases stocked prawns represented 2% or less of the catch and only two cases exceeded 10% recapture rate (Hamasaki and Kitada, 2006). However, some other stock enhancement programs for *Penaeus japonicus* and *P. chinensis* reported high recovery rates (stocked animals were identified by tags and uropod clipping) varying from 4% to 35.6% (Liu, 1990; Loneragan et al., 2006; Wang et al., 2006). A pilot stock enhancement program conducted for eastern king prawn in South-Eastern Australia identified contribution levels up to 50%, however this contribution was calculated based on very small sample size (*n* = 27) (Setio, 2016). The high recapture rate may be attributed to the estuary (Lake Tyers) being closed to the ocean, preventing stocked prawns emigrating out the mouth (Setio, 2016) highlighting how stock enhancements can benefit fisheries in such situations.

Reliable methods for identification of hatchery individuals at recapture is crucial for the precise assessment of stock enhancement programs. For some prawn release programs, stocked animals have been identified using coded wire tags and uropod clipping, as in Japan (Hamasaki and Kitada, 2006), and some enhancement programs were assessed simply considering harvest yield changes/gains (Davenport et al., 1999). Genetic marker technologies have been used in many finfish stock enhancement programs, such as to estimate the contribution stocked fish in recreational or commercial catches (e.g., Taylor et al., 2021) and to trace the origin of fish where hatchery information is incomplete or not available (Hsu et al., 2020). Different types of genetic markers are being used for population and pedigree analyses, from DNA microsatellite markers to single nucleotide polymorphisms (SNPs) (Premachandra et al., 2017; Premachandra et al., 2019; Knibb et al., 2020), but provided there is substantial diversity, we have found the use of mitochondrial haplotype sequences is often cost effective and practical (McMillen-Jackson and Bert, 2004; Premachandra et al., 2017). Moreover, as mitochondrial DNA is matroclinously inherited, we can often infer family linages (Niwa et al., 2003; Cothran et al., 2005; Knibb et al., 2014b). To date, genetic detection technology is comparatively rare for detecting released crustaceans except a few cases reported for crabs and prawns (Obata et al., 2006; Setio, 2016; Wang et al., 2016; Liu et al., 2018).

A 2-year release program for eastern king prawn was initiated in recruitment limited estuaries in Australia in 2014, to enhance recreational fisheries for the species. The associated monitoring program collected prawn samples and assessed abundance from stocked and unstocked estuaries and this statistical information was reported to provide an initial non genetic determination of the potential contribution of the releases to prawn populations within the estuaries (Becker et al., 2018). The objectives of the present study were to, first, use the above collected samples, and further samples, to assess the genetic structure of the wild eastern king prawn populations collected from four estuaries from Queensland/NSW border to Southern NSW before stock enhancement. Our second objective was to test for signals of the hatchery-released animals during post-release monitoring in the stocked estuaries using genetic measures, namely mitochondrial control region sequence.

## 2 Materials and method

### 2.1 Prawn stocking and sample collection

**FIGURE 1:**
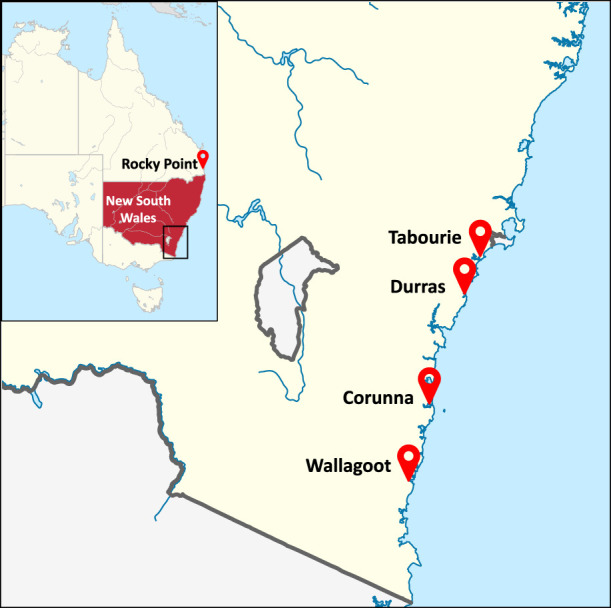
Geographical locations of eastern king prawn sample collected estuaries in New South Wales and Rocky Point hatchery in Queensland, Australia. Durras and Corunna estuaries were non-stocked reference systems, and Wallagoot and Tabourie estuaries were stocked with hatchery releases. Maps available in Wikimedia Commons web pages (https://commons.wikimedia.org/wiki/File:New_South_Wales_in_Australia.svg and NordNordWest/Wikipedia; "https://commons.wikimedia.org/wiki/File:Australia_New_South_Wales_location_map_blank.svg" \o "https://commons.wikimedia.org/wiki/File:Australia_New_South_Wales_location_map_blank.svg"https://commons.wikimedia.org/wiki/File:Australia_New_South_Wales_location_map_blank.svghttps://commons.wikimedia.org/wiki/File:Australia_New_South_Wales_location_map_blank.svg) were adapted under the Creative Commons license (https://creativecommons.org/licenses/by-sa/3.0/de/deed.en).

Wild broodstock consisted of female prawns that had already received spermatophores. They were collected by commercial trawlers and transported to hatchery facilities at Rocky Point (Figure 1). In the hatchery, females spawned naturally, or in some cases spawning was induced by eyestalk enucleation (Kelemec and Smith, 1980). Prawns were reared to post-larvae size (∼18 mm TL) over 20 days before being transported to the release locations. Stocking occurred in two estuaries during December of 2014 and again in December of 2015 (See Table 1 for release locations and numbers) with greater numbers released during 2015.

**TABLE 1:** The number of eastern king prawns stocked and sampled by year of released and estuary.

Estuary	Treatment	2014 stocking	2015 stocking	Samples taken
Durras	References	0	0	168
Corunna	References	0	0	170
Tabourie	Stocked	260,000	1,305,000	234
Wallagoot	Stocked	726,000	3,501,000	253

Four estuaries (Figure 1) consisting of two non-stocked reference systems (Durras and Corunna) and two stocked systems (Tabourie and Wallagoot) were sampled nocturnally for prawns using a cast net “Fitech, Memphis, TN, USA, 2.45 m diameter, 4.75 mm square monofilament mesh”. Eastern king prawn were collected during multiple sampling trips from all estuaries other than Wallagoot prior to the first 2014 stocking event (Supplementary Table S1). No prawns were sampled from Wallagoot as we understood that the estuary had not received a natural recruitment event in several years and none could be collected despite intensive sampling efforts. After the 2014 stocking, multiple sampling trips were made to each of the estuaries until October 2016 (Supplementary Table S1), aided by citizen-science sampling programs to collect prawns as they grew, resulting in between 168 and 253 samples per estuary (Table 1). Wallagoot estuary remained closed to the ocean after the 2014 stocking, meaning eastern king prawn samples collected before October 2015 (time when the estuary did open to the sea), were all suspected to be of hatchery origin. In addition to the prawns sampled from the estuaries, 46 wild broodstock were also collected from the Rocky Point hatchery facilities that were used for the 2015 stocking event (no broodstock were retained prior to the 2014 stocking). However, the 46 broodstock may not have represented the complete set of broodstock for the 2015 event and an unknown portion were lost. Tissue samples were placed into 2 ml vials containing 85% ethanol and stored at 4 °C until used for genomic DNA extraction.

### 2.2 DNA extraction and PCR amplification of mtCR

Genomic DNA extraction was conducted using QIAGEN DNeasy Blood and Tissue Kit, following manufacture’s protocol. The integrity of extracted DNA was tested using 1% agarose gel electrophoresis. PCR amplification of mtDNA control region (mtCR) was achieved using a published primer pair (Chan et al., 2014). PCR assays were conducted in 30 µL reaction volumes containing, 0.2 µM of each primer (forward 5′-ATTAGCACTAGGTACTGAGA-3′and reverse 5′-AGT​TTC​AGG​ATA​AGA​AGA​CAC​TAT-3′) in 2 μL, 15 µL of 2 × PCR Master Mix, 11 µL of RNase free water, and 2 µL of the DNA template. Amplification was performed using an Eppendorf Mastercycler Nexus (Hamburg, Germany) with pre-denaturation at 94°C for 5 min; 35 cycles of denaturation at 94°C for 30 s, annealing at 55°C for 30 s, extension at 72°C for 50 s; and final extension at 72°C for 10 min. Amplicons were tested on 1.5% agarose gels stained with ethidium bromide to ensure successful amplification.

DNA sequencing of PCR products was conducted using DNA Sanger sequencing (AGRF, Australia) from both directions using the specific PCR primer pair mentioned above. Sequences were evaluated for the sequencing errors where there was disagreement between the forward and reverse sequences, the electropherograms were inspected manually, or when one of the two sequences corresponded to the consensus sequence, the consensus sequence was used.

### 2.3 Genetic diversity and population structure analysis

Genetic diversity within and among populations was estimated using mtCR sequences. Sequences were aligned (ClustalW) and tested for DNA sequence similarity within and between sample locations using BioEdit Sequence alignment editor V7.0.5.3 (Hall, 1999). Haplotype diversity, nucleotide diversity, number of polymorphic loci and presence of private alleles were tested using DnaSP v.5.0 (Librado and Rozas, 2009) and/or GenAIEx V6.5 (Peakall and Smouse, 2012). Genetic diversity among population were tested using PhiPT estimates, an F_ST_ analogue which calculates population differentiation based on the genotypic variance (Teixeira et al., 2014) and analysis of molecular variance (AMOVA) among populations was conducted using GenAIEx V6.5 (Peakall and Smouse, 2012). Phylogenetic analysis for mtCR sequences was carried out implementing Maximum Likelihood method. Prior to the construction of phylogenetic tree, an assessment was conducted to find the best nucleotide substitution model and GTR + G + I (General Time Reversible + Gamma distribution + evolutionary invariable) model was selected as the best-fitting nucleotide substitution model for the maximum likelihood analysis based on Akaike Information Criterion (AIC) and Bayesian Information Criterion (BIC) scores using MEGA 11 (Tamura et al., 2021). The pattern of genetic relationship among samples was created using principal coordinate analysis (PCoA) test available in GenAlEx V6.5 (Peakall and Smouse, 2012) based on the haploid genetic distance matrix to examine any population structure due to geographical proximity. The first two axes explaining the majority of the variance among the samples were used to obtain the PCoA plot.

## 3 Results

### 3.1 Genetic diversity and similarities among sites

#### 3.1.1 Basic descriptions on mtCR haplotype and allelic variation

A stretch of 716 bp sequence fragment was recovered from 871 samples. Considering all samples, 294 polymorphic sites were detected along the 716 bp length, and a total of 750 mtCR haplotypes were identified among 871 samples and five populations (GenBank accessions ON804899—ON805769). The average haplotype diversity (*h*) and average nucleotide diversity (*π*) was 99.8% and 2.3%, respectively.

Considering all samples from the different times of sampling (all 871 samples collected across both years), each separate population had at least some specific types of haplotypes shared between individuals within that given population (Supplementary Figure S1A), with the notable exception of wild-collected broodstock sampled from Rocky Point Hatchery, where every sample had a different (i.e., unique), haplotype. When sharing occurred it was at very low level for Durras (of 167 different haplotypes recorded, only one was shared i.e., one haplotype was in more than one animal) and also low at Corunna (168 and two, respectively) and a little higher in Tabourie (227 and seven, respectively) and highest in Wallagoot (168 and nine, respectively). Considering sharing among different populations, most populations shared some haplotypes with each other, albeit at low levels (a minimum of 0 to a maximum of 6 haplotypes, Supplementary Figure S1B).

#### 3.1.2 Statistical analyses comparing sites and times of sampling

**FIGURE 2:**
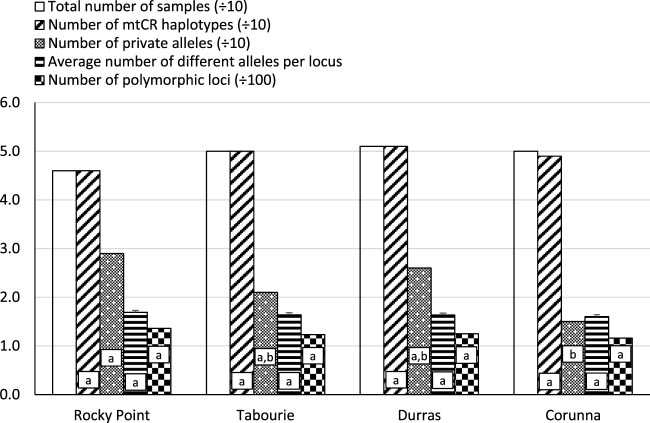
The number of mtCR haplotypes and allelic pattern distribution among four eastern king prawn populations. The number of mtCR haplotypes is the number of different/unique mtCR sequences found in a given population. The number of private alleles is the number of mtCR alleles (at a given nucleotide position) that occurred only in one population and not in others. The average number of different alleles is the number of different alleles at a given nucleotide loci, averaged over all nucleotide loci. The number of polymorphic loci is the number of nucleotide positions which were polymorphic (i.e., that had more than one nucleotide base). The analyses were conducted using samples collected before release from each site, and sample sizes were close to 50 for each site (Supplementary Table S1). No samples from Wallagoot were available before stocking. Bars with the same letters are not significantly different form each other (*p* > 0.05). For mtCR haplotype counts, number of private alleles and number of polymorphic loci, significance levels were determined using **(A)** Chi-square tests based on total counts and **(B)** using the Bonferroni method to correct for multiple comparisons. For average number of different alleles, significance levels were assessed using **(A)** ANOVA and **(B)** Bonferroni post hoc test for multiple comparisons.

There was a total of 44 different collections made over the four estuaries, at different times, both before and after stocking. Excluding the data from the stocked sites after stocking, pairwise PhiPT analyses of each sample at each time indicated there were 20 significant cases out of a total of 210 comparisons at *p* < 0.05 (Supplementary Table S2). Due to the relatively low level of significant cases (only a little above that expected by chance) and their temporally inconsistent nature, we have pooled samples from estuaries across the multiple sampling times prior to stocking. After pooling the temporal samples there were 195 haplotypes for the total of 197 samples. Statistical tests comparing between estuaries using the samples collected before release, indicated most measures of genetic diversity did not significantly vary among estuaries (Figure 2).

#### 3.1.3 Tests of population genetic structure

Pairwise comparison of PhiPT values for samples collected before stocking did not indicate any significant genetic structure among populations (Table 2). All the PhiPT values were <0.01 or close to zero. AMOVA test also did not identify any significant differences among populations (PhiPT = 0.001; *p* = 0.429) and all the variation (100%) was attributable to within population variation. DNA sequence divergence estimates were very similar for within populations and among populations comparisons, varying between 2.25%–2.29% (Table 3).

**TABLE 2:** Pairwise population differences measured using PhiPT values among four eastern king prawn populations using samples collected before stocking. PhiPT values are shown below diagonal, and the statical significance of these values are (probability, based on 999 permutations) shown above diagonal. No samples from Wallagoot were available before stocking. *n* = sample sizes.

	Rocky point	Tabourie	Durras	Corunna
Rocky point (*n* = 46)	—	0.397	0.448	0.414
Tabourie (*n* = 50)	0.000	—	0.092	0.456
Durras (*n* = 51)	0.000	0.008	—	0.229
Corunna (*n* = 50)	0.000	0.000	0.003	—

**TABLE 3:** Mean percent mtCR DNA sequence divergence for within and between four eastern king prawn populations, using samples collected before stocking. The percent divergence, for a given data point in the table, is calculated as the divergence in nucleotide sequence between pairs of samples, averaged over all pairs of samples. Mean percent divergence for within populations is shown in shaded cells along the diagonal. No samples from Wallagoot were available before stocking. *n* = sample sizes.

	Rocky point	Tabourie	Durras	Corunna
Rocky point (*n* = 46)	2.29	—	—	—
Tabourie (*n* = 50)	2.29	2.26	—	—
Durras (*n* = 51)	2.28	2.27	2.26	—
Corunna (*n* = 50)	2.29	2.25	2.25	2.28

**FIGURE 3:**
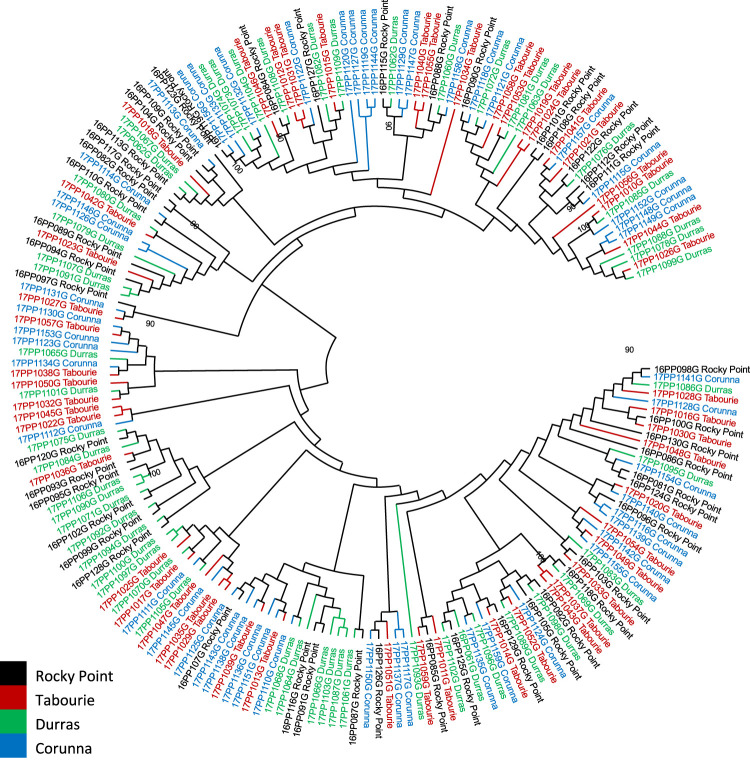
Phylogenetic analysis of the eastern king prawn mtCR sequences from four populations collected before stocking. No samples from Wallagoot estuary were available before stocking. Analysis was conducted using maximum likelihood method using GTR + G = I model in MEGA11 (Tamura et al., 2021). Each sample is colour coded based on the sample location, as indicated within the image. Only bootstrap values >90 are included as text in the figure (bootstrap value >90 offer statistical support for the grouping of the samples).

Phylogenetic (Maximum-Likelihood tree) analysis of the mtCR sequences generally did not support division of samples based on the populations (Figure 3) with some exceptions involving animals from Wallagoot (after stocking) where some similar haplotypes were grouped together with high bootstrap support values (Supplementary Figure S2). PCoA analysis reveals no genetic structure among the populations from various locations and the genetic relatedness among the samples did not form any distinct cluster based on locations (Supplementary Figure S3).

### 3.2 Evidence for contribution of hatchery-releases

#### 3.2.1 Haplotype sharing within sites

**FIGURE 4:**
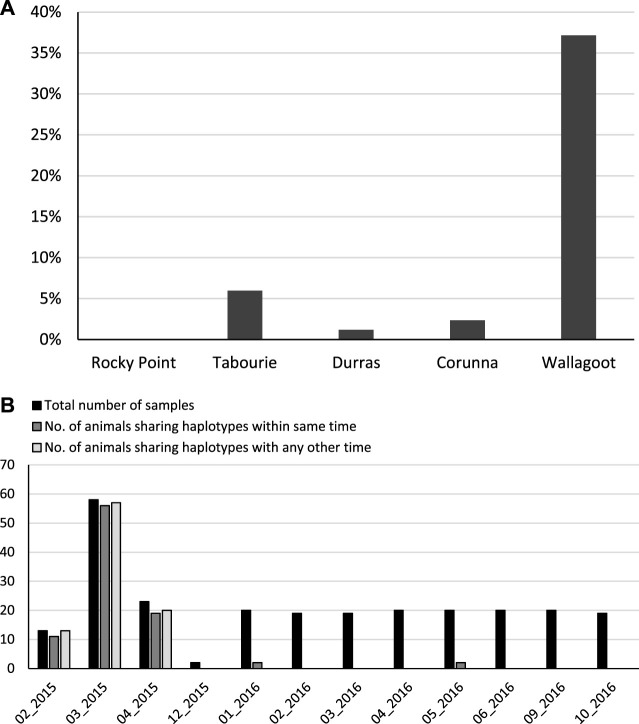
The level of mtCR haplotype sharing between animals within populations. **(A)** considering all populations and all samples, bars represent the percentage of animals that shared mtCR haplotypes within each population. **(B)** considering only the Wallagoot site, bars represent the number of animals sharing mtCR haplotypes by sample collection time.

For the unstocked estuaries Corunna and Durras, nearly every animal had its own unique mtDNA haplotype, specifically 99% and 98% of haplotypes were unique respectively. By contrast, only 63% of the samples from Wallagoot, and 94% of the samples from Tabourie had unique mtDNA haplotypes (Figure 4A). A total of 94 samples out of the total of 253 samples shared haplotypes at Wallagoot, consisting of 9 shared haplotype groups. Specifically, over 80% of the samples collected within the first 5 months after the 2014 release in Wallagoot had shared haplotypes (Figure 4B).

Pairwise PhiPT values estimated considering the different sample collection time cohorts indicated that the early collections of Wallagoot samples within the first 5 months of stocking were significantly different from the later samples (Supplementary Table S2). Considering standard number of samples per estuary (i.e., *n* = 46) selected randomly irrespective of the sample collection time from each site, Wallagoot samples had the lowest number of haplotypes among all the populations (Supplementary Figure S4), and the individuals sharing the same haplotypes were grouped together with high bootstrap support values in the phylogenetic analysis (Supplementary Figure S2).

#### 3.2.2 Detection of “rocky point” hatchery haplotypes in other sites

As a further indication of a stocking signal, we tested the presence of matching haplotypes between hatchery and other populations. Considering all the samples, only the two stocked estuaries had some individuals with the same haplotypes as those found in the hatchery broodstock individuals but at low frequencies (Supplementary Figure S5). Specifically, these matching haplotypes were found in samples collected after the second stocking event (when we had samples from some broodstock), except for one individual from Tabourie that was sampled before stocking.

## 4 Discussion

### 4.1 Genetic diversity

The observed haplotype diversity for eastern king prawn was very high, especially in the unstocked reference estuaries where more than 99% of animals had a unique haplotype. Previous work has shown similar patterns in diversity, with private mtCR haplotypes across an entire sample of banana prawn *Fenneropenaeus merguiensis* (Knibb et al., 2014b) and pink shrimp *Farfantepenaeus duorarum* (McMillen-Jackson and Bert, 2004). Various factors could contribute to this high level of variation, including the likelihood of very high effective population sizes of wild prawns, and also hypervariability of the mtCR, relative to coding genes (Stoneking, 2000; Diniz et al., 2005). Furthermore, the mtCR has an apparent high mutation rate of 19%/MY, as reported for the brown shrimp (McMillen-Jackson and Bert, 2003). Indeed, McMillen-Jackson and Bert (2004) suggested that mtCR in penaeids might be a useful genetic marker. Also, the high proportion of unique haplotypes may indicate the presence of large number of different maternal families within the spawning populations along the East coast of Australia. This assumption is supported by the recent stock assessment report for eastern king prawn, that indicated a large spawning biomass (Helidoniotis et al., 2020).

Despite this high level of haplotype diversity (which can be due to a single nucleotide change over about 700 bp), there was a high level of DNA similarity across the ∼700 bp with an average of 98% similarity for all sample pairs. DNA sequence divergence between sites were very similar to the within site divergence. Consequently, most of the tests between sites (using pre-release samples and pooling the different temporal samples at each site) were not statistically significant. None of the pairwise comparison of PhiPT values were significant between sites for pooled temporal data, and 100% of the molecular variation was attributable to within population variation. Moreover, neither phylogenetic analysis, nor PCoA analysis identified segregation of samples by populations.

Collectively, these results suggest that the eastern king prawn populations in the east coast of Australia can be considered as a single panmictic genetic population, a conclusion which agrees with previous considerations based on adult migration, spawning patterns and dispersal of larvae along the coast (Montgomery, 1990; Montgomery et al., 2007; Taylor and Johnson, 2021), and also unpublished preliminary genetic work from a Ph. D. thesis (Chan, 2015). The combination of a Northward adult migration to spawning grounds (Ruello, 1975; Taylor and Johnson, 2021) and dispersal of larvae in Southwards flowing currents, likely explain the lack of any genetic structuring with the eastern king prawn population. Although there is likely to be annual variation in larval transport owing to changes in the conditions of the East Australian Current, modelling has shown the mean dispersal distances can range between ∼750 and 1,000 km before settlement (Everett et al., 2017). This provides significant opportunity for mixing to occur during the southward phase of their life history, which would contribute to the lack of structure observed in this study.

### 4.2 Evidence of contribution of released prawns

We posit that, given the high level of haplotype diversity, that shared haplotypes within sites result from kinship (i.e., individuals being members of the same families). For Wallagoot, a stocked site, a very large number of samples (∼37%), share a haplotype with at least one other sample. Indeed, within about the 6 months of the first stocking for Wallagoot, when the estuary was closed to the ocean, there is genetic evidence that nearly all samples were from stocked animals, based on the criteria of sibship. Approximately 6% of samples at Tabourie shared haplotypes with at least one other sample from this site. However, for the unstocked sites the respective values were very low (∼1% Durras and ∼2% for Corunna), which provides an estimate of natural background sharing. Given background sharing is around 1%, the values for the stocked sites could guide our estimates of the upper range of contribution rates for released prawns. Since eastern king prawn do not reproduce within estuaries, and rely upon a marine migratory phase to complete their life-cycle (Montgomery et al., 2007), isolation and co-ancestry can be excluded as possible causes for haplotype sharing. Our conclusion that shared haplotypes are due to kinship is further supported by the high level of haplotype sharing (more than 80%) occurring shortly after the first release. Pairwise comparison of PhiPT values for the temporal data indicated a significant difference for Wallagoot samples collected immediately after the release, which aligns with the high level of haplotype sharing shortly after the first release. Thus, the significant PhiPT values are assumed to arise from the inclusion of large family groups that leads to population differentiation (Wang, 2018). Finally, statistically fewer haplotypes at Wallagoot compared with the other estuaries reinforces the hypothesis of a high level of kinship at this site.

It is noteworthy that eastern king prawn collected from stocked estuaries following the second release event, when broodstock samples were available, haplotypes that were found within the hatchery broodstock were also detected in the stocked estuaries, although they were detected at low frequencies (1%–2%). The low detection rate may be due to the unknown number of missing broodstock samples. Moreover, it has been reported there is a high variance in prawn fecundity between females in the hatchery (Knibb et al., 2014a), so it could be that the females contributing most to the stocked post-larvae were not present within the sample set. Importantly, by using inferred kinship groups, contributions of stocked animals to wild populations can still be determined despite the lack of genetic information from the broodstock (also seeTaylor et al., 2021).

There was a substantial difference in the proportion of shared haplotypes (and therefore assumed kinship and stocking signal), between Wallagoot and Tabourie. In particular, over the 5 months following the 2014 release, over 80% of eastern king prawn collected from Wallagoot appear to be stocked. This strong signal at Wallagoot can be attributed to the long-term closure of the estuary to the sea, creating a type of marine lake, limiting the emigration of the animals out to sea and eliminating natural recruitment and dilution of the proportion of restocked animals into the system. Given this, releasing prawns in similar closed estuaries may be more successful than releasing in more open estuaries.

Our results for Wallagoot highlight the potential impacts of stocking in severely recruitment limited estuaries, where stocked eastern king prawn can provide benefits to anglers for at least 5 months post-release, and these findings align with previous surveys of pilot release in these systems (Taylor, 2017). The other stocked site, Tabourie, maintained connectivity with the ocean leading up to both stocking events, probably allowing natural recruitment of wild eastern king prawn into the system. However, we still observed elevated levels of shared haplotypes (6%) at Tabourie compared to the reference estuaries (1%–2%). If we correct the proportion of eastern king prawn with shared haplotypes for background levels estimated in the reference estuaries, it appears the stocking could have contributed up to 5% of animals within this system.

Collectively, these findings show that eastern king prawn releases contribute to estuarine fisheries even where natural recruitment is occurring. Last, our results demonstrate that given the likely absence of broodstock information, the kinship approach is an effective method of identifying stocked animals.

Lastly, on the basis of our results, we reflect on the efficiency and cost effectiveness of molecular tagging vs. classical tagging for stock enhancement programs, especially those for crustacea. Historically, released fish/individuals were detected using different chemical markers, for example, otolith marking supported monitoring for black bream *Acanthopagrus butcheri* (Cottingham et al., 2020) and mulloway *Argyrosomus japonicus* (Taylor et al., 2021), or physical tags (elastomer tagging, coded wire tags) for trout species (*Salmo* and *Oncorhynchus* spp.) and red snapper *Lutjanus campechanus* (Hale and Gray, 1998; Brennan et al., 2007). For monitoring of released crustacea, these aforementioned historical methods are probably inappropriate because crustacea moult (losing any potentially stained or marked exoskeleton) and/or are too small during larval and post larval stages to accept wire tags/elastomer tagging. During the last decades, genetic tagging approaches have been used increasingly for monitoring the origin, family lines and kinship of the released animals (any species and any life stages) due to a range of advantages/considerations, not the least that individuals do not need to be tagged *per se* (they carry their own genetic tags) (Taylor et al., 2021). Bravington and Ward (2004) assessed the utility of DNA microsatellite genotyping and statistical approaches in stock enhancement programs for brown tiger prawns *P. esculentus*. The use of DNA microsatellites is still valid today, although newer cost equivalent methods such as SNPs are widely available (Premachandra et al., 2019). Should there be a high level of diversity for mitochondrial haplotypes (as we found for eastern king prawns), then use of mtDNA sequences can be a more cost effective but less technically demanding option than DNA microsatellites or SNPs, and indeed we found that mtDNA haplotypes well informed the present study as to the success of the prawn releases.

## 5 Conclusion

The present study reports several novel results and conclusions from an attempted prawn stock enhancement program (which in itself is a rather novel undertaking) and also novel results from population genetic assessments. Firstly, we conclude nearly all wild samples have unique mtDNA haplotypes, perhaps reflective of large population sizes. Second, notwithstanding the diversity, we conclude no significant differences among populations suggesting a panmictic stock along the NSW coast. Third, to compensate for incomplete broodstock records and samples, we used a novel approach of comparing the level of haplotype sharing to infer restocking success assuming shared haplotypes likely indicate siblings. The restocked estuaries had much higher frequencies of shared haplotypes than the unstocked estuaries suggestive of a stocking signal. Lastly, we conclude that the strongest stocking signal was at a location isolated from the sea, effectively a type of lake, and this novel knowledge may assist further stocking programs.

## Data Availability

The datasets presented in this study can be found in online repositories. The names of the repository/repositories and accession number(s) can be found below: https://www.ncbi.nlm.nih.gov/genbank/, ON804899–ON805769.
