# Supplementary material for: Eastern king prawn Penaeus plebejus stock enhancement—Genetic evidence that hatchery bred prawns have survived in the wild after release
Source: Front Genet. 2022 Nov 8;13:975174. doi: 10.3389/fgene.2022.975174 (PMC9679433; doi:10.3389/fgene.2022.975174)
Supplement: Supplementary file 1 [file DataSheet1.docx]

# Supplementary material

**Eastern king prawn** ***Penaeus plebejus* stock enhancement – genetic evidence that hatchery bred prawns have survived in the wild after release**

H.K.A. Premachandra^a,^ *, Alistair Becker^b^, Matthew D. Taylor^b^, Wayne Knibb^a, c^

^a^ Centre for Bioinnovation, University of the Sunshine Coast, 90 Sippy Downs Drive, Sippy Downs, Qld 4556, Australia.

^b^ Port Stephens Fisheries Institute, New South Wales Department of Primary Industries, LockedBag1, Nelson Bay, NSW 2315, Australia.

^c^ Currently at Aquabreed Pty Ltd.

* Corresponding author. Email address: ahoranek@usc.edu.au

Figure S1. The number of different types of eastern king prawn mtCR haplotypes shared between more than one animal A) within each population and B) between populations. The analyses were conducted using all 871 samples collected across both years, and all possible site combinations are shown for figure 2B. Durras and Corunna estuaries were non-stocked reference systems, and Wallagoot and Tabourie estuaries were stocked with hatchery releases.

Figure S2. Phylogenetic analysis of mtCR sequences from five populations that includes all 871 samples. Analysis was conducted using maximum likelihood method and GTR+G=I model in MEGA11 (Tamura et al., 2021). Each sample is colour coded based on the sample location, as indicated within the image. Only bootstrap values > 90 are included in the figure.

Figure S3. PCoA plot of the first two axes that explained the majority of genetic variance among the individuals. This dataset included samples that were collected before stocking and no samples from Wallagoot were available before stocking.

Figure S4. Allelic pattern and haplotypes distribution among populations for standard number of samples (*n* = 46).

Footnote: The analyses were conducted using standard number of samples from each site. Bars with the same letters are not significantly different form each other (P > 0.05). For the reduced number of samples (i.e. 46 x 5 = 230), there were 217 polymorphic loci and 216 haplotypes. For mtCR haplotype counts, number of private alleles and number of polymorphic loci, significance levels were determined using (a) Chi-square tests based on total counts and (b) using the Bonferroni method to correct for multiple comparisons. For average number of different alleles, significance levels were assessed using (a) ANOVA and (b) Bonferroni post hoc test for multiple comparisons.

Figure S5. Number of haplotypes shared with Rocky Point hatchery population.

Footnote: Here, all the samples were considered for the analysis. However, about 30% more samples were available from the stocked sites than the non stocked sites and after standardizing the samples to lowest number (i.e. 168 samples at Durras) we still recovered two matching haplotypes at Tabourie and three at Wallagoot.

Table S1. Information on samples collected at different time cohorts.

| Population | Status | Date of Sampling/ time cohort | No. of Samples sequenced |
| --- | --- | --- | --- |
| Rocky Point | Hatchery | NA | 46 |
| Tabourie | Before stocking | 07_2014 | 7 |
|  |  | 09_2014 | 5 |
|  |  | 11_2014 | 38 |
|  | After stocking | 12_2014 | 20 |
|  |  | 04_2015 | 18 |
|  |  | 08_2015 | 19 |
|  |  | 01_2016 | 20 |
|  |  | 02_2016 | 20 |
|  |  | 03_2016 | 20 |
|  |  | 04_2016 | 20 |
|  |  | 05_2016 | 20 |
|  |  | 06_2016 | 2 |
|  |  | 08_2016 | 6 |
|  |  | 10_2016 | 19 |
| Durras | Before stocking | 07_2014 | 21 |
|  |  | 09_2014 | 19 |
|  |  | 11_2014 | 11 |
|  | After stocking | 12_2014 | 20 |
|  |  | 04_2015 | 18 |
|  |  | 08_2015 | 20 |
|  |  | 03_2016 | 20 |
|  |  | 05_2016 | 20 |
|  |  | 10_2016 | 19 |
| Corunna | Before stocking | 09_2014 | 28 |
|  |  | 11_2014 | 22 |
|  | After stocking | 12_2014 | 19 |
|  |  | 04_2015 | 20 |
|  |  | 08_2015 | 20 |
|  |  | 03_2016 | 20 |
|  |  | 05_2016 | 20 |
|  |  | 10_2016 | 20 |
| Wallagoot | After stocking | 02_2015 | 13 |
|  |  | 03_2015 | 58 |
|  |  | 04_2015 | 23 |
|  |  | 12_2015 | 2 |
|  |  | 01_2016 | 20 |
|  |  | 02_2016 | 19 |
|  |  | 03_2016 | 19 |
|  |  | 04_2016 | 20 |
|  |  | 05_2016 | 20 |
|  |  | 06_2016 | 20 |
|  |  | 09_2016 | 20 |
|  |  | 10_2016 | 19 |

Table S2. Pairwise population PhiPT values based on sample location, sample date and restocking status.

PhiPT Values are shown below diagonal. Probability based on 999 permutations is shown above diagonal. ‘Before’ and ‘After’ indicates sampling time before or after stocking activity. Probability values < 0.05 are indicated on bold.
